# Connection between oxidative stress and subcellular organelle in subarachnoid hemorrhage: Novel mechanisms and therapeutic implications

**DOI:** 10.1111/cns.14348

**Published:** 2023-07-05

**Authors:** Jiahao Zhang, Zeyu Zhang, Xiaoyu Wang, Yibo Liu, Qian Yu, Kaikai Wang, Yuanjian Fang, Cameron Lenahan, Maohua Chen, Sheng Chen

**Affiliations:** ^1^ Department of Neurosurgery, The Second Affiliated Hospital, School of Medicine Zhejiang University Hangzhou China; ^2^ Clinical Research Center for Neurological Diseases of Zhejiang Province Hangzhou China; ^3^ Department of Neurosurgery, Renji Hospital, School of Medicine Shanghai Jiao Tong University Shanghai China; ^4^ Center for Neuroscience Research Loma Linda University School of Medicine Loma Linda California USA; ^5^ Department of Neurosurgery, Wenzhou Central Hospital Affiliated Dingli Clinical Institute of Wenzhou Medical University Wenzhou China

**Keywords:** antioxidant, Nrf2, oxidative stress, subarachnoid hemorrhage, subcellular organelle

## Abstract

Spontaneous subarachnoid hemorrhage (SAH) is one of the most devastating forms of stroke, with limited treatment modalities and poor patient outcomes. Previous studies have proposed multiple prognostic factors; however, relative research on treatment has not yet yielded favorable clinical outcomes. Moreover, recent studies have suggested that early brain injury (EBI) occurring within 72 h after SAH may contribute to its poor clinical outcomes. Oxidative stress is recognized as one of the main mechanisms of EBI, which causes damage to various subcellular organelles, including the mitochondria, nucleus, endoplasmic reticulum (ER), and lysosomes. This could lead to significant impairment of numerous cellular functions, such as energy supply, protein synthesis, and autophagy, which may directly contribute to the development of EBI and poor long‐term prognostic outcomes. In this review, the mechanisms underlying the connection between oxidative stress and subcellular organelles after SAH are discussed, and promising therapeutic options based on these mechanisms are summarized.

## 1 INTRODUCTION

Subarachnoid hemorrhage (SAH) remains one of the most devastating forms of stroke, with high mortality and disability rates throughout the world.^1^ It is a medical emergency accounting for approximately 5% of all stroke cases. However, 50% of the survived patients suffer from permanent disability, with an estimated lifetime cost of more than double that of an ischemic stroke.^2^ Early research has determined that cerebral vasospasm (CVS) following SAH constitutes one of the most severe complications in terms of patient morbidity and mortality.^3^, ^4^ However, not all vasospasms lead to clinical changes.^4^ Moreover, mortality rates and clinical outcomes were not improved by decreasing the vasospasm rate in clinical trials.^5^ Therefore, this suggests that other pathophysiological factors, independent of angiographic vasospasm, contribute to poor clinical outcomes. Brain injury after SAH is divided into two stages: (1) early brain injury (EBI) caused by transient global ischemia and toxic constituents in the blood and (2) delayed brain injury caused by CVS. EBI has been described as an evolving frontier in SAH research.^6^, ^7^

Oxidative stress is one of the main mechanisms of EBI following SAH. Cerebral ischemia and ischemia/reperfusion (I/R) induce a series of biochemical and cellular reactions, generating in excess of reactive oxygen species (ROS). ROS are short‐lived and highly reactive small molecules, such as superoxide (O_2_^−^), hydrogen peroxide (H_2_O_2_), and hydroxyl radicals (OH^−^).^8^ Under physiological conditions, the body constantly produces ROS, which is counteracted by an antioxidant system to maintain the dynamic balance of its lower levels. However, either the excessive ROS production or the disruption of the antioxidant system after SAH results in a significant disruption of the cerebrovascular system and neuronal network.^8^ The accumulation of ROS also damages endothelial cells leading to the disruption of the blood–brain barrier (BBB).^9^

The major subcellular organelles in the central nervous system (CNS) are the nucleus, endoplasmic reticulum (ER), mitochondria, and lysosomes. Numerous studies have shown that numerous subcellular organelles, including the mitochondria, ER, and lysosomes, are functionally altered post‐SAH, and contribute to certain aspects of EBI, such as oxidative stress.^10^, ^11^

In this review, the connection between oxidative stress and subcellular organelles after SAH is summarized, and relative therapeutic strategies are proposed.

## 2 OXIDATIVE STRESS AFTER SAH

In a state of homeostasis, the ROS produced by the cells and the antioxidant substances (e.g., glutathione and catalase) produced by the antioxidant system is in a dynamic balance. However, this balance is disrupted after SAH, resulting in the oxidative system's dominance over the antioxidant system in the body. This eventually leads to the occurrence of oxidative stress. Although it has been suggested that oxyhemoglobin and its metabolites are the primary sources of ROS during SAH pathophysiology,^12^ heme also has the ability to react with hydrogen peroxide to generate hydroxyl radicals through the Fenton reaction. Nonetheless, it is generally accepted that the main sources of ROS are mitochondria and NADPH oxidase (NOX).^13^ Mitochondria are known to produce ROS under physiological conditions.^14^ With the occurrence and development of SAH, brain tissue in the perfused area of the ruptured blood vessel suffers ischemic damage, causing a fatal blow to the mitochondria in the cells in this area.^15^ Mitochondrial damage (including changes in mitochondrial membrane permeability, disruption of mitochondrial complexes, and disruption of the oxidative respiratory chain) ultimately leads to the overproduction of ROS.^16^ It has been shown that the NOX enzyme family appears to be the main contributor to oxidative stress after ischemia.^9^ ROS production by the CNS's NADPH oxidase under physiological conditions is necessary for normal brain functions, such as neurogenesis and intercellular signaling.^17^ The NADPH oxidase family consists of the following seven members: NOX1 through NOX5, DUOX1, and DUOX2.^18^ This can be further divided into three groups. The first group includes NOX1‐3, the second group includes NOX5, DUOX1, and DUOX2, and the third group has NOX4.^18^ The first group consists of the NOX subunit and the protein p22phox, in which the latter is capable of stabilizing the NOX subunit when they bind to specific cytoplasmic proteins (e.g., p47phox), they form the complete NADPH oxidase, which in turn generates ROS.^18^, ^19^ The third group only requires p22phox and not the cytoplasmic activators, and produces mainly hydrogen peroxide (H_2_O_2_) rather than superoxide anions.^18^, ^19^ In contrast, superoxide compounds produced by other pathways need to be further reduced to hydrogen peroxide (H_2_O_2_) by mitochondrial superoxide dismutase 2 (SOD2).^20^ Calcium ions activate the second group, which has a calcium‐sensing EF structural domain.^18^ There are numerous studies that have shown increased expression of NOX2 and ROS after reperfusion following cerebral ischemia.^21^ Zhang et al. found that the levels of NOX2 and NOX4 were significantly increased in neurons and astrocytes around the hematoma.^22^ Kim et al. found that NOX can induce delayed cerebral vasospasm in an animal model of SAH.^23^

Oxidative stress is an essential mechanism of EBI after SAH. At the cellular level, oxidative stress can damage phospholipids, proteins, nucleic acids, and other macromolecules, causing cellular death, vascular endothelial injury, and BBB disruption.^24^ Oxidative stress can also initiate apoptosis by promoting cytochrome c release, increasing p53, and activating caspase‐3.^25^, ^26^ Animal experiments have proven that some antioxidants, including astaxanthin, dimethyl fumarate, and tert‐butylhydroquinone, can alleviate EBI after SAH by inhibiting oxidative stress.^27^, ^28^, ^29^ In addition, oxidative stress may play an essential role in the development of EBI by interacting with subcellular organelles,^10^ which will be described below.

## 3 CONNECTIONS BETWEEN OXIDATIVE STRESS AND SUBCELLULAR ORGANELLES AFTER SAH

### 3.1 Oxidative stress and mitochondria

Mitochondrial complex I is the predominant source of ROS in mitochondria.^30^ Under physiological conditions, an intricate antioxidant defense system balances the physiological ROS generation to avoid oxidative stress caused by mitochondria.^30^, ^31^ For instance, superoxide dismutase can convert superoxide radicals to hydrogen peroxide, which is then further detoxified by catalase.^30^ However, when various stimuli, such as hypoxia and ischemia, prompt a sudden production of an excessive amount of reactive oxygen species, exceeding the scavenging ability of the antioxidant defense system, the body will be placed in a state of oxidative stress.^32^

For complex I to remain intact, the proportion of reduced flavin mononucleotide (FMN) is thought to be crucial and determined by the NADH/NAD^+^ ratio. Therefore, an increase in the NADH/NAD^+^ ratio and O_2_^−^ formation is often present in the events such as injury, ischemia, etc.^33^ Complex I produces O_2_^−^ in the presence of NADH, and this production is enhanced by the complex I inhibitor rotenone.^34^ After SAH, brain cells are in a hypoxic state with a high NADH/NAD^+^ ratio. This leads to the inactivation of various dehydrogenases and a decreased metabolism of NAD^+^ compared to normoxic conditions.^35^ After hypoxia, the altered NADH/NAD^+^ ratio is not the result of a single action. First, the accumulation of sulfide in the brain after hypoxia leads to an increased NADH/NAD^+^ ratio.^36^ When respiration is impaired, serine catabolism then leads to NADH accumulation.^37^ Moreover, the disruption of the electron transport chain after hypoxia can also lead to the accumulation of NADH at the source. In addition to this, the reduction of the coenzyme Q pool also leads to the interruption of the electron transport chain, resulting in the inability of the electrons taken off from NADH to pass downstream, and the occurrence of electron leakage to bind to oxygen at complex I to form superoxide anions.^38^ Hypoxia and calcium overload caused by SAH‐induced cerebral ischemia affect electron transport and promote the mitochondrial release of excess ROS.^39^ Exogenous CoQ supplementation partially restored HIF‐1α degradation and ROS production.^40^ Both of the pathways mentioned above generate ROS at the IF (FMN site) site, whereas the IQ (CoQ binding site) site generates ROS under particular circumstances.^41^ In respiratory complex I, the site IQ generates ROS at high rates during reverse electron transfer of ubiquinol in the ubiquinone/ubiquinol pool, which is supported by high proton motive force across the inner mitochondrial membrane.^42^

**FIGURE 1:**
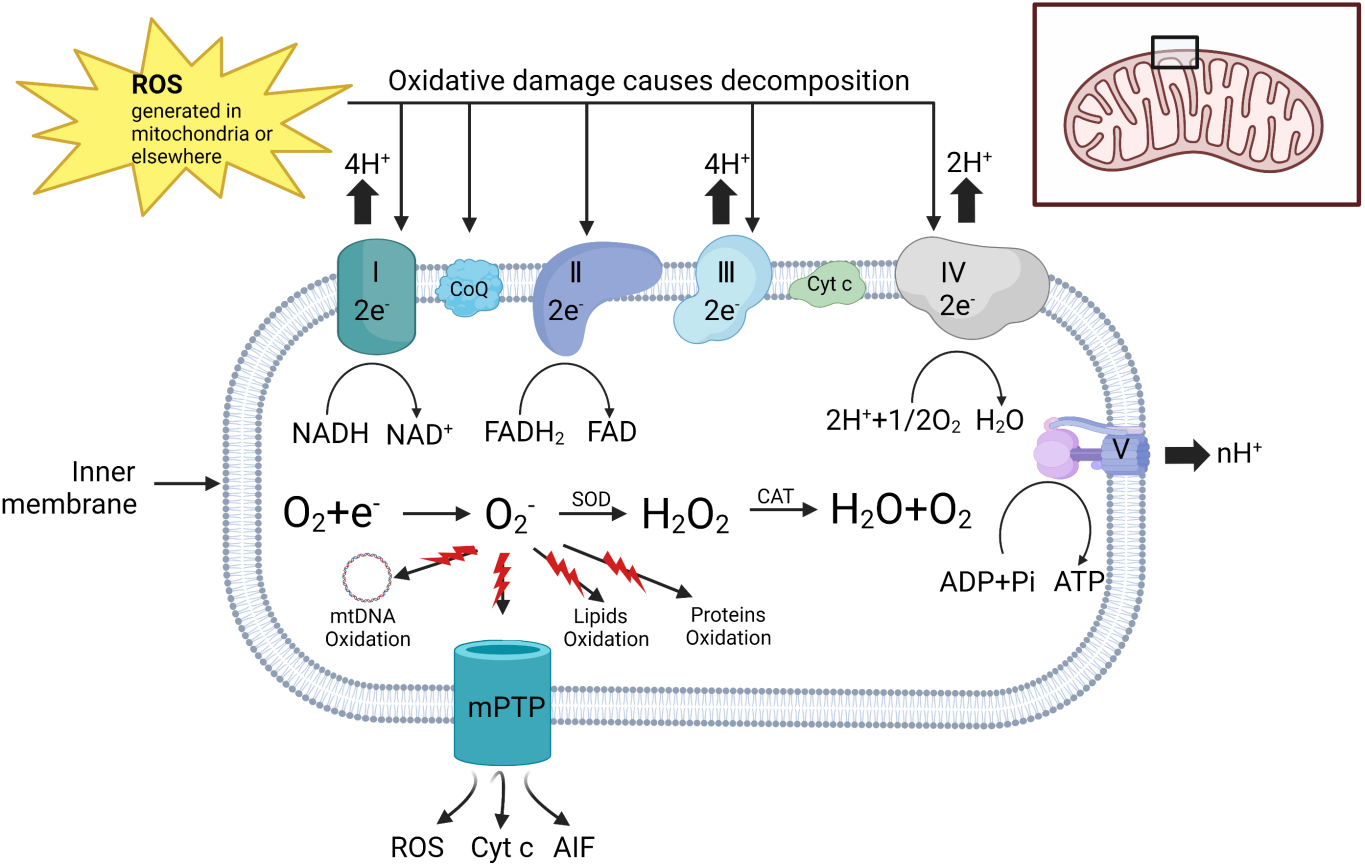
The relationship between mitochondria and oxidative stress. Under normal circumstances, electrons will be transferred between complexes I–IV. The mitochondrial respiratory chain (including complex and CoQ) is disrupted by reactive oxygen species generated in mitochondria or elsewhere inside and outside the cell, initiating the degradation process. Once the complex or CoQ is destroyed, the electrons will escape, and O_2_^−^ will be generated after contact with oxygen. O_2_^−^ can be removed by the antioxidant system under normal circumstances, but after SAH, the antioxidant system is destroyed. Antioxidants are excessively depleted and various enzymes that promote the production of antioxidants (e.g., glutathione reductase) and various antioxidant enzymes (e.g., peroxide dismutase) are oxidatively damaged, denatured, or even degraded. Excessive accumulation of O_2_^−^ causes damage to mitochondrial DNA, lipids, and proteins. It also keeps mPTP open, allowing ROS and Cyt c to overflow into the mitochondria. AIF, apoptosis‐inducing factor; mPTP, mitochondrial permeability transition pore; ROS, Superoxide dismutase.

The SAH‐induced whole brain ischemia‐induced hypoxia and calcium overload affects the electron transport chain and promotes the release of excess ROS from mitochondria. Excessive release of ROS can directly lead to the destruction of mitochondrial membrane structure and mitochondrial DNA. The disrupted mitochondria generate and release more ROS, creating a positive feedback. Mitochondria are disrupted after SAH and produce ROS in large quantities, while the antioxidant system cannot clear the overproduced ROS in a timely manner. The mitochondria often then become targets of ROS with fatal consequences. Since most of the ROS produced by mitochondria appear first in the matrix, mitochondrial proteins, membranes, and mitochondrial DNA (mtDNA) are the most frequently disrupted components.^43^ ROS also disrupts various membrane structures, leading to altered mitochondrial permeability. Furthermore, Increased ROS can mediate sustained opening of the mitochondrial permeability transition pore (mPTP), and prolonged mPTP opening can lead to ROS bursts and ROS‐induced ROS release (RIRR). At the same time, this is often accompanied by a burst of nitric oxide production and ROS‐induced release of reactive nitrogen oxides (RNS).^44^ The sustained ROS‐mediated opening of mPTP also leads to the release of cytochrome c and apoptosis‐inducing factor (AIF) from mitochondria into the cytoplasm (Figure 1).^45^ Cytochrome c binds to apoptotic protease‐activating factor 1 (APAF1) in the cytoplasm to form apoptotic bodies, and Lys72 helps to stabilize this interaction. Subsequently, apoptotic vesicles activate caspase‐9, which further de‐activates caspase‐3 leading to apoptosis.^46^ Caspase‐3 can also cleave the anti‐apoptotic BCL2 family member BCL‐xL to produce a product that promotes mitochondrial outer membrane permeabilization (MOMP).^47^ This suggests the existence of a caspase‐3‐mediated amplification loop. AIF is an apoptosis mediator that is not dependent on cystathionine, which is excreted from the mitochondria and translocated to the nucleus, leading to the production of DNA fragments that further lead to cell death.^48^, ^49^ Cyclosporin A inhibits the opening of mitochondrial mPTP and reduces the efflux of cytochrome and AIF, thereby improving brain injury after SAH.^50^ In addition, the mitochondrial permeability shift leads to the release of OMI and a second mitochondria‐derived cysteine aspartase activator (SMAC). The release of these substances blocks the cysteine inhibitor X‐linked apoptosis inhibitory protein (XIAP) and thus promotes apoptosis.^51^

### 3.2 Oxidative stress and ER

The ER plays a vital role in many normal cellular physiological functions, such as the “packaging” of newly synthesized proteins, post‐translational modifications, and maintenance of intracellular Ca^2+^ homeostasis.^52^, ^53^ Once the internal environment of the ER is changed, such as changes in the redox state and changes in the level of Ca^2+^, it will bring great pressure to the ER and cells. This stress leads to impaired ER function, including its predominant protein folding capacity, leading to further accumulation of misfolded/unfolded proteins and ultimately to ER stress and activation of unfolded protein response (UPR).^54^ The original purpose of ER stress is to promote cell survival, but it can also have the effect of being “overdone” once excessive ER stress leads to cell death.^55^ In experimental SAH, marked ER swelling and ER stress were found to mediate cortical neuronal apoptosis.^56^ SAH results in global cerebral ischemia, which has been shown to severely impact ER function, leading to ERS, and activation of the UPR.^57^

**FIGURE 2:**
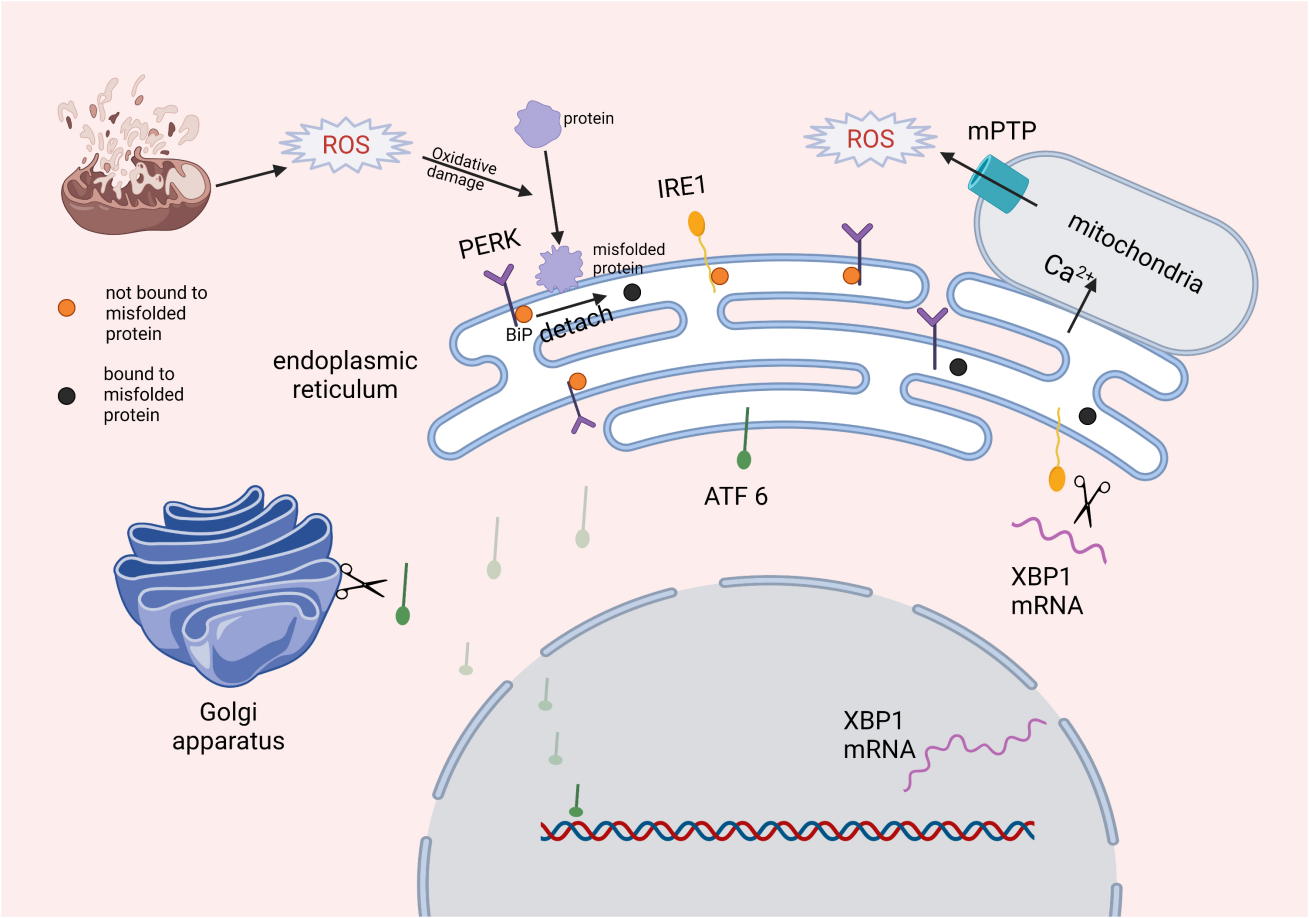
The relationship between the endoplasmic reticulum and oxidative stress. The surface of some mitochondria is directly connected to ER, which is rich in IP3R, and Ca^2+^ can directly enter mitochondria during oxidative stress. There are three main signaling pathways of ER stress: PERK, IRE1, and ATF6. Commonly, PERK and IRE1 are linked to Bip, and their activity is inhibited. When oxidative stress occurs, oxidative damage leads to a significant increase in misfolded proteins. Bip binds misfolded proteins (yellow balls represent Bip not bound to misfolded protein and black balls represent Bip bound to misfolded protein), resulting in a conformational change and detachment from PERK and IRE1, so that its inhibitory effect on PERK and IRE1 disappears. Both IRE1 and PERK undergo oligomerization and autophosphorylation upon separation from BiP, and ATF6 is translocated to the Golgi for further processing. Activated PERK can inhibit protein translation, activated IRE1 can cleave XBP1 mRNA, and ATF6 can translocate into the nucleus to promote XBM1 mRNA transcription. ATF6, activating transcription factor 6; IRE1, inositol‐requiring enzyme 1; mPTP, mitochondrial permeability transition pore; PERK, pancreatic ER kinase (PKR)‐like ER kinase; ROS, Superoxide dismutase; XBP1, X‐box protein 1.

The ER also produces ROS, while the ER oxidoreductase ERO1α forms a disulfide bond to counteract ER stress. However, transferring electrons to molecular oxygen creates ROS as a by‐product.^58^ The pro‐apoptotic transcription factor, CHOP, induces ERO1α expression.^59^ In an animal experiment, apelin‐13 alleviated EBI after SAH via suppression of ER stress‐mediated apoptosis and BBB disruption through the ATF6/CHOP pathway.^60^ ER stress can induce Ca^2+^ release through the ERO1α/inositol 1,4,5 triphosphate receptor (IP3R) pathway.^61^, ^62^ It is estimated that 5%–20% of the mitochondrial surface and ER are tightly connected (Figure 2).^63^ The contact site between mitochondria and ER is enriched with IP3R, where Ca^2+^ can enter the mitochondria directly from the ER.^63^ Under ER stress, Ca^2+^ transfer from ER to mitochondria is increased.^64^ The enrichment of Ca^2+^ in mitochondria leads to a large and continuous opening of mPTP,^65^ and the consequences of such have been discussed previously. In addition, mitochondrial ROS leads to the oxidation of cysteine residues of ranibulin receptors (RyRs; another ER Ca^2+^ release channel),^66^ resulting in alterations in their gating and Ca^2+^ sensitivity.^66^ In all, ER stress may exacerbate EBI after SAH by promoting oxidative stress.

Reduced global cerebral blood flow due to SAH results in tissue hypoxia and hypoglycemia, both of which rapidly induce protein misfolding and ER stress. Reperfusion of affected tissue causes oxidative stress when blood flow is restored.^55^ ROS produced by the ER or other pathways after SAH can lead to protein misfolding or improper modification, thereby leading to ER stress. The main role of ER stress is to inhibit the translation of new proteins and promote the refolding of unfolded/misfolded proteins, helping the cell to survive.^55^ There are three main signaling pathways of ER stress: pancreatic ER kinase (PKR)‐like ER kinase (PERK), inositol‐requiring enzyme 1 (IRE1), and activating transcription factor 6 (ATF6) (Figure 2).^67^ The common feature of these three ER stress sensors is that they all contain an ER luminal domain. PERK and IRE1 are type 1 transmembrane proteins, and PERK and IRE1 bind to the ER chaperone BiP, which inhibits their activity.^68^ When BiP binds misfolded proteins, BiP dissociates from PERK and IRE1 as a way to initiate their activation.^69^ Additionally, the misfolded protein can act as a ligand to directly bind the luminal structural domain of IRE1 and activate IRE1.^70^ IRE1α has two enzymatic activities‐serine/threonine kinase structural domain and a ribonucleic acid endonuclease (RNase) structural domain.^71^ The RNase of IRE1 cleaves the mRNA encoding the XBP1 (X‐box protein 1) transcription factor to generate the steady‐state transcription factor XBP1, which then translocates to the nucleus to induce the transcription of many genes, thereby increasing the function of the ER.^72^ Activated PERK phosphorylates eukaryotic translation initiation factor 2 subunit α, thereby hindering ribosome assembly and reducing protein synthesis.^73^ This gives cells the opportunity to try to fold unfolded/misfolded proteins. ATF6 is a type 2 transmembrane protein that translocates to the Golgi apparatus and is cleaved by Site‐1 and Site‐2 proteases to activate in response to ER stress.^68^ The activated ATF6 translocates to the nucleus, induces the transcription of XBP1 mRNA, and XBP1 mRNA can be spliced and modified by the activated IRE1, ultimately enhancing ER function.^72^ Moderate ER stress is beneficial for cell survival, whereas if ER stress remains high for a long time, the UPR program can self‐destruct.^55^ In SAH, inhibition of ER stress and oxidative stress may alleviate EBI and neuronal death. One study showed that resveratrol exerted a neuroprotective effect on SAH by reducing oxidative damage, ER stress, and neuroinflammation.^74^ Therefore, reducing oxidative damage to ER after SAH can keep ER stress in a moderate range, which is beneficial to cell survival.

### 3.3 Oxidative stress and nucleus

**FIGURE 3:**
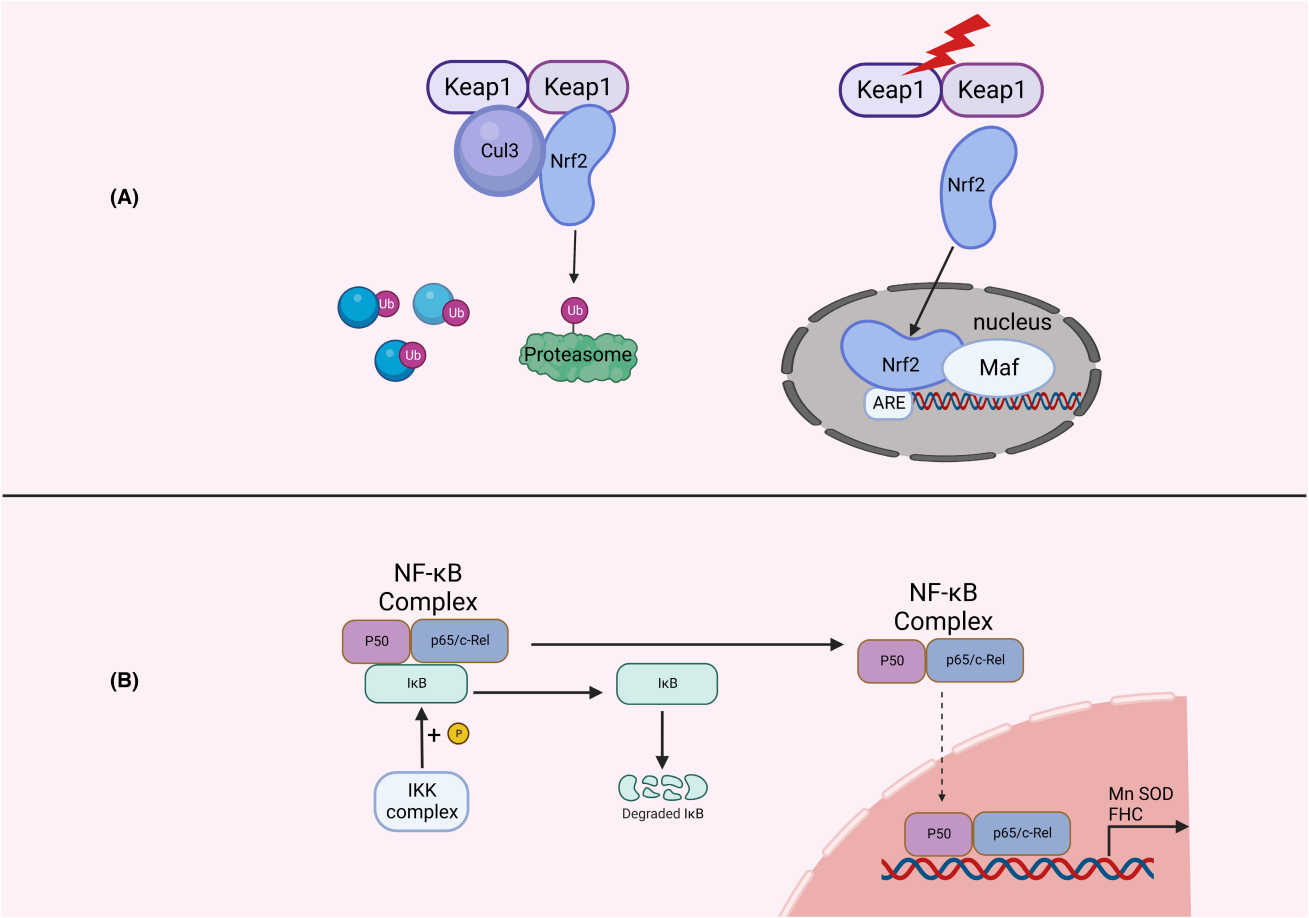
The relationship between the nucleus and oxidative stress. (A) When the keap1 dimer binds to Nrf2, Cul3 can simultaneously bind to it and recruit the proteasome (green) to ubiquitinate Nrf2, maintaining its low intracellular concentration. When the keap1 dimer is oxidatively damaged, Nrf2 is released and further translocated into the nucleus to form a complex with Maf. Complex acts on antioxidant response elements (ARE). (B) Physiologically, IκB binds the dimer of p50 and p65 and restricts its entry into the nucleus. The IKK complex is phosphorylated when oxidative stress occurs to degrade IκB further. The dimers of p50 and p65 are translocated into the nucleus and promote the production of antioxidants such as MnSOD and FHC. ARE, antioxidant element; FHC, ferritin heavy chain; IKK, IκB kinase; Keap1, Kelch‐like ECH‐associated protein 1; NF‐κB, nuclear factor κB; Nrf2, Nuclear Factor‐Erythroid 2‐Related Factor 2.

The nuclear factor‐erythroid 2‐related factor 2 (Nrf2) signaling (Figure 3A) —Nrf2 is a cap “n” collar (CNC) transcription factor with a leucine zipper structure in the basic region.^75^ Keap1 is a redox‐sensitive E3 ubiquitin ligase substrate junction that regulates intracellular Nrf2.^76^ Nrf2 is a protein with seven Nrf2‐ECH homology domains (Neh1‐7), in which Neh2 interacts with two Keap1 molecules through two binding sites, the ETGE motif and DLG motif, in which the former has a significantly more potent effect than the latter.^77^ Under normal conditions, because Keap1 is an adaptor protein for the Cul3 E3 ubiquitin ligase,^78^ the half‐life of Nrf2 is very short, resulting in the amount of Nrf2 being maintained at a low level. The burst of oxidative stress after SAH results in the oxidative inactivation of Keap1 and the cessation of Nrf2 ubiquitination, accumulating in the cytoplasm and further translocating to the nucleus.^79^ Nrf2 then forms heterodimers with members of the small Maf protein family (MafF, MafG, and MafK).^80^ The Nrf2‐small Maf complex binds to the antioxidant response element (ARE) in the promoters of cytoprotective genes, leading to the upregulation of the expression of related proteins, such as NAD(P)H: quinone oxidoreductase 1 and proteins used in the synthesis of glutathione.^81^ The Keap1‐Nrf2‐antioxidant response element (ARE) pathway has been found to be one of the most important defense mechanisms against oxidative stress.^82^ Studies have proven that some antioxidants, including astaxanthin, dimethyl fumarate, and tert‐butylhydroquinone, can alleviate EBI after SAH by activating this pathway.^27^, ^28^, ^29^

NF‐κB signaling (Figure 3B)—The NF‐κB transcription factor family consists of five members: RelA (p65), RelB, and c‐Rel, and the precursor proteins NF‐κB1 (p105) and NF‐κB2 (p100), which are processed into p50 and p52, respectively. All NF‐κB proteins share a Rel homologous structural domain responsible for DNA binding and dimerization.^83^ At cellular homeostasis, NF‐κB dimers bind to IκB proteins, thereby sequestering the NF‐κB complex in the cytoplasm. Phosphorylation of the IκB kinase (IKK) complex degrades IκB proteins, leading to their translocation into the nucleus.^83^ Manganese superoxide dismutase (MnSOD) is the most important protein with an antioxidant activity whose expression is promoted by NF‐κB.^84^ MnSOD can convert superoxide anion into hydrogen peroxide, which is further decomposed into H_2_O and O_2_. NF‐κB can also promote the translation of ferritin heavy chain (FHC); although, FHC does not directly eliminate ROS, it can inhibit iron‐mediated Fenton reaction.^85^ In fact, the Fenton reaction after SAH is no different from other types of brain injury in terms of its chemical reaction process. It is the reaction between Fe^2+^ and hydrogen peroxide to generate Fe^3+^ and hydroxyl radicals. However, the amount of bleeding after SAH is larger than that of TBI and cerebral hemorrhage, and more Fe^2+^ is released after the rupture of red blood cells. Therefore, after a large amount of heme is exposed, the Fenton reaction is particularly violent. In addition to its antioxidant effect, NF‐κB can also promote the generation of ROS, which has opposite consequences. NF‐κB can induce the expression of gp91phox to regulate phagocytosis of NADPH oxidase.^86^ As mentioned above, NADPH oxidase can generate ROS. NF‐κB causes inducible nitric oxide synthase iNOS (NOS2) or neuronal nitric oxide synthase nNOS (NOS1).^87^, ^88^ In fact, NO itself is not involved in oxidative stress, but it can react with superoxide to generate peroxynitrite. Peroxynitrite can cause a variety of cellular damage, including protein nitration, lipid peroxidation, and mitochondrial dysfunction.^89^ In all, NF‐κB signaling plays an important role in EBI after SAH, and has been shown to be a therapeutic target for SAH in many studies.^90^, ^91^, ^92^, ^93^

### 3.4 Oxidative stress and lysosome

The lysosome, long known as the “cellular recycling center,” is a membranous structure with a lumen filled with various hydrolytic enzymes that are mainly used to break down large molecules of organelles, including lipids, polysaccharides, and proteins.^94^ Lysosomes have phagocytosis and autophagy capacities, which are important for maintaining the homeostasis of cells and material recycling. ROS can cause irreversible damage to the lysosomal membrane. It can increase lysosomal permeability by inducing cross‐linking of lysosomal membrane proteins through disulfide bonds,^95^ causing the release of various hydrolases originally isolated in intercalated compartments into the cytoplasm, leading to cell destruction.^96^ However, moderate oxidative stress activates the pro‐survival function of lysosomes. Oxidative stress activates chaperone‐mediated autophagy, and the susceptibility of oxidized proteins to be taken up by lysosomes is greatly increased.^97^ It has also been demonstrated that macroautophagy can discriminately remove damaged mitochondria during oxidative stress, in which the transition of mitochondrial permeability is a key event in initiating mitophagy.^98^ The close relationship between oxidative stress and autophagy alludes to another possibility for the treatment of SAH, as autophagy activation after SAH has been demonstrated in a large body of literature. Lee and his partners have demonstrated the activation of the autophagic pathway after SAH. In their study, microtubule‐associated protein light chain 3 (LC3), cathepsin D, and beclin‐1 were investigated by western blot analysis and immunohistochemistry.^99^

## 4 RELATED ANTIOXIDANT STRATEGIES FOR SAH TREATMENT

Excessive production of ROS due to the burst of oxidative stress after SAH can damage various organelles, and may further lead to cell death. Antioxidant therapy is expected to be a treatment strategy for SAH. Antioxidant therapy has two apparent approaches, reducing ROS production and increasing ROS clearance. Researchers generally favor the latter as a treatment prospect.

### 4.1 Increasing ROS scavenging

Superoxide dismutase (SOD) is the only enzyme that can scavenge O_2_^−^ in mammalian cells. Therefore, its replacement therapy has been regarded as the antioxidant therapy with the most potential, and many SOD mimics have been invented. Manganese porphyrins are the most widely studied SOD mimetics. Among which, MnTM‐2‐pYp5+ and MnTE‐2‐pYp5+ showed very high SOD activity. Their improved prognostic effect on stroke has also been demonstrated in rat models of cerebral ischemia, subarachnoid hemorrhage, and in vivo studies.^100^, ^101^, ^102^ In computer simulations, GC4419 is a selective antioxidant that selectively removes superoxide anions without reacting with other oxidants.^103^ The compound also has a significant effect on the treatment of cancer.^104^ Although GC4419 has not been tested in humans in SAH, it has been shown to have a significant effect on mucosal stomatitis induced by chemoradiotherapy in head and neck cancer. Moreover, its structure also suggests that it can play an antioxidant role in SAH. Ebselen, a glutathione peroxidase mimetic, has been shown to reduce oxidative damage and prevent neuronal death in both animal experiments and clinical trials of aneurysmal subarachnoid hemorrhage.^105^, ^106^, ^107^ As mentioned above, activation of Nrf2 due to oxidative modification of the keap1 protein can lead to upregulation of the expression of numerous antioxidant‐related proteins, which makes activation of Nrf2 an effective therapeutic tool. Songorine, a typical active C20‐diterpene alkaloid from the lateral root of Aconitum carmichaelii, can promote the degradation of Keap1 to activate Nrf2.^108^ Songorine has been shown to ameliorate myocardial damage in a mouse model of septic heart injury by enhancing antioxidant effects, and its effects in the brain have also been demonstrated in mice. Unfortunately, no current studies verified the antioxidant effect of Songorine in SAH and its effect on prognosis, yet it has the potential to become a new direction for research. Mitoquinone, a mitochondria‐targeted antioxidant, can activate mitophagy through the Keap1/Nrf2/PHB2 pathway, thereby inhibiting oxidative stress‐related neuronal death in rats SAH model.^109^ A study on SAH in rats by Liu et al. showed that Arachidonyl‐2‐chloroethylamide (ACEA) could promote mitochondrial autophagy to attenuate oxidative stress and neurological dysfunction by promoting the CB1R/Nrf1/PINK1 pathway.^110^ Bakuchiol (Bak) was shown to increase SOD and GSH‐Px activity and attenuate EBI after SAH by attenuating BBB disruption, oxidative stress, and apoptosis by modulating Trx1/TXNIP expression and AMPK phosphorylation.^111^ The effect of Bak on improving the prognosis of SAH has only been confirmed in the mouse SAH model, and clinical research still focuses on its anti‐aging effect on the face.^112^ In the rat SAH model, SS31 was shown to restore GPx and SOD activity, thereby reducing brain edema, improving neurological deficits and neuronal apoptosis.^113^

### 4.2 Reducing ROS generation

Despite previous discussion, it has also been argued that therapeutic means of scavenging peroxides using exogenous molecules are impractical because the rate of ROS production due to the burst of oxidative stress after the onset of SAH is not matched by the rate of exogenous antioxidant scavenging. One study showed that scavenging OH^−^ is impractical because all organic compounds react with OH^−^ at similar rate constants close to the diffusion limit, and the only way to prevent its harmful effect is to prevent its production.^114^ The mitochondrial pathway and the NADPH pathway are the two main pathways of ROS production, as discussed previously. Hence, Limiting ROS production in these two pathways emerges as a potential therapeutic target. There are two main mechanisms of NOX inhibitors, either through the inhibition of enzyme activity or the inhibition of enzyme assembly. In addition to the ROS scavenging effect mentioned above, Ebselen can also inhibit the subunit assembly of NOX2 to inhibit the generation of ROS.^115^ Although OP2113 is not a conventional antioxidant drug, it can act as a specific inhibitor of ROS mitochondrial pathway production.^116^ The action site of OP2113 is the IQ site of complex I of the mitochondrial respiratory chain, and it does not affect mitochondrial energy metabolism while inhibiting the production of ROS.^116^ Although this result was obtained in a rat myocardial infarction model, we agreed that its antioxidant mechanism is highly likely to be replicated in SAH. Therefore, we think that OP2113 also has the prospect of treating SAH. In a SAH rat model, heat shock protein 22 (hsp22) exerts neuroprotective effects by rescuing mitochondrial function in an AMPK‐PGC1α‐dependent manner and also regulates TFAM/Nrf1‐induced mitochondrial biogenesis and DRP1‐triggered mitochondrial apoptosis, further reducing oxidative stress and brain injury.^117^ Fang et al. demonstrated that PACAP can reduce mitochondria‐mediated oxidative stress and neuronal apoptosis after subarachnoid hemorrhage by knocking out the pituitary adenylate cyclase‐activating polypeptide (PACAP) gene and adding exogenous PACAP38 in rat SAH model.^118^ Zhang et al. demonstrated that puerarin not only inhibited SAH‐induced ROS production but also increased SOD activation after SAH in the mouse SAH model.^119^

A comprehensive list of compounds that can exert antioxidant effects in vivo and further reduce brain damage after SAH, is outlined in Table 1.

**TABLE 1:** Compounds that can exert antioxidant effects and their mechanisms.

Compound	Effect	Citations
MnTE‐2‐pYp^5+^	Mimetic of SOD, reacts with ROS to eliminate its oxidation	[100, 101, 102]
GC4419	Selective removal of superoxide anions without reacting with other oxidants	[103]
Ebselen	glutathione peroxidase mimetic inhibit the subunit assembly of NOX2 to inhibit the generation of ROS	[105, 106, 107, 115]
Songorine	Promote the degradation of Keap1 to activate Nrf2	[108]
Mitoquinone	Activate mitophagy	[109]
ACEA	Activate mitophagy	[110]
Bakuchiol	Increase SOD and GSH‐Px activity	[111, 112]
SS31	Restore GPx and SOD activity	[113]
OP2113	Specific inhibition of mitochondrial production of ROS	[116]
Hsp22	Eescue mitochondrial function	[117]
PACAP38	Reduce mitochondrial‐mediated oxidative stress	[118]
Puerarin	Inhibit ROS production Increased SOD activation	[119]

Abbreviations: ACEA, Arachidonyl‐2‐chloroethylamide; Hsp22, heat shock protein; NOX, NADPH Oxidases; PACAP, pituitary adenylate cyclase‐activating polypeptide; ROS, reactive oxygen species; SOD, Superoxide dismutase.

## 5 CONCLUSION AND PERSPECTIVE

SAH is a condition with high rates of mortality and disability, but treatment options remain limited. Although a great deal of research has been conducted on SAH, the mechanisms that contribute to its poor prognosis remain unclear, which greatly limits the development of treatments for SAH. Nonetheless, abundant evidence demonstrates that oxidative stress plays an important role in the pathophysiological process of EBI after SAH, causing damage to cellular and subcellular structures. In the study of oxidative damage to subcellular organelles, mitochondria, nucleus, ER, and lysosomes are the hot topics of research and the most likely potential targets for SAH treatment. In the antioxidant treatment of SAH, increasing ROS clearance and reducing ROS production both have shown therapeutic potential. However, there is still a long way to go in the clinical application of these treatment strategies. Therefore, further exploration is necessary to broaden our understanding of the relationship between oxidative damage and various subcellular organelle dysfunction after SAH, and it plays a role in the pathogenesis of SAH. It is paramount in further promoting the development of therapeutic strategies for SAH.

## AUTHOR CONTRIBUTIONS

JHZ, ZYZ, XYW, YBL, and KKW conceptualized the study. SC and MHC carried out supervision. JHZ, ZYZ, XYW, YBL, and QY performed visualization. JHZ, ZYZ, XYW, YJF, and QY carried out writing—original draft preparation. JHZ, ZYZ, XYW, YBL, CL, QY, and SC carried out writing—review and editing. All authors have read and agreed to the published version of the manuscript.

## FUNDING INFORMATION

This study was supported by the National Natural Science Foundation of China (81971107, to SC).

## CONFLICT OF INTEREST STATEMENT

The authors declares that there is no conflict of interest regarding the publication of this paper.

## Data Availability

All the data supporting the findings of this study are included in this article.
